# Multi-antibiotic resistant brain abscess sensitive only to chloramphenicol: a case report

**DOI:** 10.4076/1757-1626-2-6352

**Published:** 2009-08-25

**Authors:** Atiq ur Rehman, Tausif Rehman, Rushna Ali

**Affiliations:** 1 Department of Neurosurgery, King Fahd Specialists HospitalAl-QassimSaudi Arabia; 2 Department of Neurosurgery, University of New Mexico AlbuquerqueNew MexicoUSA; 3 School of Medicine, Aga Khan UniversityP.O.Box 3500, Stadium Road, KarachiPakistan

## Abstract

**Introduction:**

A brain abscess is a focal, intracerebral infection that begins as a localized area of cerebritis and develops into a collection of pus surrounded by a well-vascularized capsule.

**Case presentation:**

An 18 year old male was diagnosed to have culture-negative bilateral subdural empyema, which was drained and the patient was discharged, only to return 3 months later with a left temporo-parietal abscess that was drained and continued to show no growth on cultures and was non-responsive to multiple antibiotics. As a final effort, chloramphenicol therapy was begun and the patient showed immediate improvement and made a relatively uneventful recovery.

**Conclusion:**

Chloramphenicol is an antimicrobial agent used rarely today in the United States because of its associated adverse effects. But the fact remains, that it is a broad-spectrum agent that is highly effective against many gram-positive and gram-negative bacteria, spirochetes, chlamydiae, and rickettsia. Due to its ability to achieve high concentrations in the cerebrospinal fluid we would advise it as second line therapy for culture negative brain abscesses.

## Introduction

Intracranial abscesses are a life-threatening condition, and include brain abscess, subdural empyema and intracranial epidural abscess. A brain abscess is a focal, intracerebral infection that begins as a localized area of cerebritis and develops into a collection of pus surrounded by a well-vascularized capsule [1]. Most brain abscesses are caused by injury or local infection, or by a focus of infection in a contiguous structure (40-50%) [2,3]. Brain abscesses can also be caused by hematogenous or metastatic spread from a distant focus of infection (25%) or can be cryptogenic (15%). Fever, headache, altered consciousness, and hemiparesis are the most common manifestations [4]. Treatment of brain abscess requires a combination of antimicrobials, surgical intervention, and eradication of primary infected foci. We describe a case of a multi-antibiotic resistant brain abscess that was found to be sensitive only to chloramphenicol.

## Case presentation

An 18-year-old Arab male from Saudi Arabia, presented with headache and vomiting. The patient was irritable, confused, and febrile. He had neck rigidity and left sided hemiplegia. Investigations revealed a high white blood cell count. CT scan and MRI of brain showed extensive subdural collection of pus bilaterally, more so on the right side.

Subdural empyema was drained through bilateral burr holes. Patient received metronidazole and ceftriaxone. Pus was cultured but showed no growth even after 21 days. The patient’s condition improved and he was discharged after 3 weeks.

Three months later he was brought to the Emergency Room with headache and altered sensorium. CT scan of brain with contrast showed a ring enhancing lesion in the left temporo-parietal region. Keeping in view his past history and radiological features of the lesion, a diagnosis of cerebral abscess was made. The abscess was approached through a burr hole and thick yellow pus was aspirated. A drain was left in the abscess cavity. As before, there was no growth on bacterial or fungal cultures.

Cefotaxime, penicillin and metronidazole were given, but fever and neck stiffness persisted. After twelve days, antibiotics were changed to metronidazole, vancomycin and amikacin. The patient continued to deteriorate and a week later ceftazidime and teicoplanin therapy was begun, without any improvement.

After five days, intravenous chloramphenicol was given for three days and then orally for another four days, after which no more chloramphenicol was available at our institution. Following this short course of chloramphenicol therapy, the patient’s fever subsided, WBC count returned to normal, and there were clear signs of clinical improvement. CT scan was repeated and showed resolution of abscess cavity.

The patient continued to improve and became conscious. He became mobile and was discharged after a total of three months in-hospital stay. A follow up in clinic three weeks later, showed almost complete neurological recovery with mild weakness on right side and a slight limp on walking.

## Conclusion

Despite the advent of modern neurosurgical techniques, new antibiotics, and new powerful imaging technologies, brain abscess remains a potentially fatal central nervous system infection. Studies have shown that penicillin, ampicillin, cefuroxime, chloramphenicol, rifampicin, trimethoprim, co-trimoxazole, cefotaxime, ceftazidime, clindamycin and metronidazole, all achieve therapeutic concentrations in brain tissue and intracranial pus [1,4]. The antibiotics of choice to be used empirically are crystalline penicillin, chloramphenicol, and metronidazole, followed by definitive therapy based on the sensitivity pattern of the causative organisms. There is a recent trend toward the avoidance of chloramphenicol [1]. Chloramphenicol is an antimicrobial agent used rarely today in the United States because of its associated adverse effects, particularly idiosyncratic aplastic anemia. At one time it was hailed as a broad spectrum agent highly effective against many gram-positive and gram-negative bacteria, spirochetes, chlamydiae, and rickettsia. Chloramphenicol had a major role in the treatment of meningitis caused by *Haemophilus influenzae*, *Streptococcus pneumoniae*, and *Neisseria meningitidis* because of its bactericidal activity against these organisms and the ability to achieve high concentrations in the cerebrospinal fluid [5].

Chloramphenicol continues to play a major role in the treatment of serious infections in developing countries and may be of great therapeutic benefit for the management of life-threatening drug-resistant infections or in persons who are allergic to penicillin [6]. It is important, therefore, that we reacquaint ourselves with this potent antimicrobial. We report this case as chloramphenicol, despite its potential side-effects, can potentially be a highly useful antibiotic in brain infections when cultures are negative and identification of the offending pathogen becomes difficult, and other antimicrobials have failed. Due to its ability to achieve high concentrations in the cerebrospinal fluid and potent bactericidal activity against a myriad of organisms we would advise it as second line therapy for culture negative brain abscesses.

## Abbreviations

CT: computed tomography
MRI: magnetic resonance imaging
